# Juvenile Huntington’s disease: two case reports and a review of the literature

**DOI:** 10.1186/s13256-020-02494-7

**Published:** 2020-10-01

**Authors:** Sigita Lesinskienė, Darja Rojaka, Rūta Praninskienė, Aušra Morkūnienė, Aušra Matulevičienė, Algirdas Utkus

**Affiliations:** 1 Clinic of Psychiatry, Institute of Clinical Medicine, Faculty of Medicine, Vilnius University, M.K. Čiurlionio 21/27, LT-03101 Vilnius, Lithuania; 2 Faculty of Medicine, Vilnius University, Vilnius, Lithuania; 3 Clinic of Children’s Diseases, Institute of Clinical Medicine, Faculty of Medicine, Vilnius University, Vilnius, Lithuania; 4 Centre for Medical Genetics, Vilnius University Hospital Santaros Klinikos, Member of the European Reference Network for Rare Neurological Diseases, Vilnius, Lithuania; 5 Department of Human and Medical Genetics, Institute of Biomedical Sciences, Faculty of Medicine, Vilnius University, Vilnius, Lithuania

**Keywords:** Juvenile Huntington’s disease, Tics, Behavior, Apathy, Family, Treatment complexity

## Abstract

**Background:**

Huntington’s disease is a rare, autosomal dominant neurodegenerative disease characterized by motor, cognitive, and psychiatric symptoms. Usually, the disease symptoms first appear around the age of 40, but in 5–10% of cases, they manifest before the age of 21. This is then referred to as juvenile Huntington’s disease. According to the small number of cases reported in the literature, the course of juvenile Huntington’s disease significantly differs from adult onset and shows significant interpatient variability, making every case unique.

**Case presentation:**

Our study aims to highlight the complexity and diversity of rare juvenile Huntington’s disease. We report cases of two Caucasian patients with chronic tics referred to the Huntington’s Disease Competence Center of Vilnius University Hospital Santaros Klinikos with suspicion of juvenile Huntington’s disease due to the appearance of chronic motor tics, and behavior problems. The diagnosis of juvenile Huntington’s disease was confirmed on both clinical and genetic grounds. In both cases described, the patients developed symptoms in all three main groups: motor, cognitive, and psychiatric. However, the first patient was experiencing more severe psychiatric symptoms; in the second case, motor symptoms (rigidity, tremor) were more prominent. In both cases, apathy was one of the first symptoms and affected patients’ motivation to participate in treatment actively. These two case descriptions serve as an important message for clinicians seeing patients with chronic tics and gradually worsening mood and behavior, indicating the need to investigate them for rare genetic disorders.

**Conclusions:**

Description of these two clinical cases of juvenile Huntington’s disease provides insight into how differently it manifests and progresses in young patients and the difficulties the patients and their families face. There were different but painful ways for families to accept the diagnosis. Because the disease inevitably affects the patient’s closest ones, it is crucial to also provide adequate psychological and social support to all the family members. Establishment of multidisciplinary specialist centers for Huntington’s disease, as demonstrated by our experience, not only allows timely diagnosis and treatment plans but also ensures thorough disease management and care for patients and systematic support for their families.

## Background

Huntington’s disease (HD; OMIM accession no. 143100, ORPHAnet no. 399) is a rare neurodegenerative disease characterized by motor, cognitive, and psychiatric symptoms [1, 2]. The condition is inherited in an autosomal dominant manner and occurs in all racial groups, with worldwide morbidity of 6–14 per 100,000 individuals [1, 3]. HD is caused by the CAG triplet expansion in *HTT* (OMIM accession no. 613004) on a short arm of chromosome 4 (4p16.3). The expanded and unstable polymorphic trinucleotide (CAG) repeat in exon 1 of the *HTT* gene is translated into an extended polyglutamine tract of the protein. An expanded allele (beyond a threshold of 35 units) through a deleterious gain-of-function mechanism leads to neuronal dysfunction and neurodegeneration. It leads to the pathogenic form of the multifunctional protein huntingtin, causing dysfunction and death of neurons in both brain and body [2–4]. The first symptoms usually appear around the age of 40, with a life expectancy of around 18 years after the appearance of the first motor symptoms [2, 5]. Due to its hereditary nature, progressive course, and changes in the patient’s behavior and personality, the disease is devastating to both the patients and their families. Despite increasing knowledge in the field, intensive study, and current clinical trials, the disease remains incurable, but it is not untreatable. Treatment focuses on maximizing patients’ quality of life through multidisciplinary management of symptoms and psychological and social support.

In 5–10% of all HD cases, the disease occurs before the age of 21; it is then called juvenile Huntington’s disease (JHD; OMIM accession no. 143100, ORPHAnet no. 248111) [1, 4, 6]. According to the few cases reported in the literature, the course of JHD significantly differs from adult-onset HD and greatly varies between patients, making every JHD case unique.

The Huntington’s Disease Competence Center of Vilnius University Hospital Santaros Klinikos (HDCC) was established in 2015 to provide multidisciplinary support to patients and families affected by the disease. As of 2019, 237 people were diagnosed with HD in Lithuania, 4 of whom had JHD. HDCC is member of the European Reference Network for Rare Neurological Diseases. Here we report two cases of patients with JHD that provide insight into how differently the disease manifests and progresses in young patients and the difficulties the patients and their families face.

## Case presentation

### Patient 1

Patient 1 was a Caucasian boy referred to a children’s neurology department in April 2017 at the age of 16 for investigation of chronic tics and worsening emotional state. The tics first appeared at the age of 11 as coughing and clearing of the throat. The tics were persistent despite treatment with tiapride hydrochloride. At the age of 15, involuntary movements of the head and neck emerged. According to the patient’s mother, over the past year, the boy had become irritable and withdrawn. He would spend most of his time alone, separated from his family and friends. School references claim that it became noticeably harder for him to concentrate in class. His ability to read, write, and calculate deteriorated. During his examination, the patient was alert and oriented to person, place, and time. His temperature, heart rate, blood pressure, respiratory rate, blood oxygen level, and blood work results were normal. His neurological examination revealed that he has constrained gait and finger tremor. A genetic disorder was suspected despite the absence of a family history.

With the informed consent of both the mother and the patient, genetic testing for HD was performed by polymerase chain reaction (PCR) analysis of the region encompassing the CAG repeat in exon 1 of the huntingtin gene (*HTT*) followed by fragment sizing through capillary electrophoresis. The results of diagnostic testing revealed a normal allele with 17 CAG repeats and expanded as well as an unstable polyglutamine-encoding allele with 52 CAG repeats. Because both positive genetic testing results and motor symptoms of the disease were present, the patient was diagnosed with JHD. Later the same year, it was discovered that HD was present in the patient’s father and his family for several generations, but the fact was kept a secret from the patient’s mother. The diagnosis was then explained to the patient and his mother, and possible medical and nonmedical treatment opportunities, as well as the follow-up plan, were discussed. 

The patient attended the HDCC six times for regular checkups and treatment. He is cared for by the multidisciplinary team, consisting of a pediatrician, a child neurologist, a child and adolescent psychiatrist, a clinical psychologist, a dietitian, a rehabilitation physician, and a social worker. Furthermore, the patient and his family members were receiving psychological and social support on an outpatient basis.

Over the course of 2 years, the patient’s disease progressed. In addition to the previously present tics, the patient developed twitching in his left shoulder, rigidity while walking, and dysarthric speech. The patient also developed difficulties with handwriting. During a checkup in 2018, new motor symptoms were observed. In essence, coordination was worse in his left arm, and left side ataxia, intentional tremor in both hands, and slightly asymmetric rigidity in the patient’s face were noted. In addition, the patient was complaining about losing strength in both arms.

The patient’s mother noticed he was becoming more emotionally labile. The patient had lost interest in communication and studies. He dropped out of school due to problems studying and attended a vocational school for a period. Eventually, it became too hard for the patient to concentrate on intellectual activity, and he stayed home most of the time. In 2017, his intelligence test showed an IQ of 64. From being apathetic and uninterested in any activity, the patient gradually became depressed. He mentioned having suicidal thoughts several times. The patient would often become anxious and physically aggressive toward his mother. There were several episodes of psychosis, which the patient described as having a strange, hard-to-describe feeling in his body. The impaired perception was observed while he was hospitalized during those episodes; the patient was having visual and auditory hallucinations.

During the patient’s hospitalizations, symptomatic treatment adjusted to his current needs was provided. Shortly after the diagnosis, he was treated with sertraline for depression. When his severe aggression outbursts and visual and auditory hallucinations occurred, haloperidol decanoate and trihexyphenidyl were prescribed. Subsequently, due to extensive side effects, his psychiatric symptoms were managed with aripiprazole and olanzapine. Because the patient’s condition was worsening, levomepromazine was added to the treatment scheme. Although the patient was still gradually losing motor and cognitive function, his aggressive outbursts, which had occurred several times per day before such treatment, became less frequent.

### Patient 2

Patient 2 was a 13-year-old Caucasian boy who was referred to the HDCC in October 2014 for HD testing. Unlike patient 1, there were two known HD cases in patient 2’s family on the paternal side. Patient 2’s mother reported gradual development of involuntary chest and hip twitching during sleep, unsteady gait, distal limb rigidity, difficulties understanding the patient’s speech, and frequent throat clearing. Similarly to the first case, behavior changes, including separation from peers and family and problems with studying, were reported. The interview revealed an episode of short-term memory loss. Rigidity in movement and occasional hyperkinesis in the hands were observed during the patient’s neurological examination. The patient was alert and oriented to person, place, and time. His temperature, heart rate, blood pressure, respiratory rate, blood oxygen level, and biochemical blood test results were normal.

With the informed consent of both the mother and the patient, genetic testing for HD was performed by PCR analysis. The results of diagnostic testing revealed a normal allele with 17 CAG repeats and an expanded and unstable polyglutamine-encoding allele with 69 CAG repeats in exon 1 of *HTT*. The patient was diagnosed with JHD.

The patient attended the HDCC three times for checkups and treatment. The patient reported progressive movement difficulties, particularly instability walking up and down stairs. The patient’s mother noticed his emotional state and cognitive abilities worsening. Teachers at his school said it had become hard to understand his writing. The patient was also experiencing difficulty in concentrating on his studies. He was becoming less interested in communicating with other people and mostly spent time playing computer games on his own. His mother reported him becoming more sensitive and impulsive. She noticed that having a visual schedule and discussing plans in advance helped them to avoid arguments at home. The patient was showing symptoms of an eating disorder due to dysphagia, and he gradually developed parkinsonism. In a 2019 evaluation, the patient’s IQ test result was at the mark of 50. Because of physical impairment, the patient needed constant support in everyday activities.

Compared with the first patient, this patient’s quality of life was mostly affected by motor symptoms of the disease. His pharmacological treatment included tiapride hydrochloride and the complex of levodopa, benserazide hydrochloride, and trihexyphenidyl, which was effective in reducing rigidity and helped the patient remain physically independent for extended periods.

### Summary

Along with the pharmacological treatments, both patients and their families were provided with psychological support. To maintain the patients’ physical and social health, they were attending physiotherapy, ergotherapy, and a youth center. In the case of patient 1, although the medical treatment was effective during hospitalization, neither pharmacological nor nonpharmacological treatments were consistently effective between visits. The team of psychologists concluded that the mother’s grief over her child’s disease was interfering with the treatment plan.

## Discussion and conclusions

We present two cases of patients with JHD diagnosed and treated at HDCC. Although both teenagers experienced their first symptoms at a similar age and first developed difficulties with studying, the disease has affected them in different ways. This demonstrates that JDH is a unique experience for every patient and requires careful examination and targeted individual treatment.

In both cases, with the well-coordinated work of experienced professionals at HDCC, the cause of symptoms was identified in the shortest possible amount of time, allowing them to proceed with an appropriate treatment strategy. Although there is no clear evidence whether accepting the uncertainty of gradually developing symptoms without knowing their cause can result in less suffering than facing the diagnosis of an incurable and progressive disease, it seems that when adequate multidisciplinary support is available, the diagnosis provides a possibility to ensure better elaborate support of quality of life for patients and their families.

The course of the disease and symptomatology described here are consistent with the literature findings on JHD. Several other cases of JHD similarly to our presented cases have been described, with the initial and main symptoms being tics: blinking, twitching of the muscles of the face and neck, snoring, or multiple tics [1, 7, 8]. It is essential to mention that the genetic results of our patients matched the clinical information. In each case, the allele was transmitted from the patient’s father, and CAG triplet expansion was greater than 50 [1, 4]. The number of triplet expansion is essential, because there is a negative correlation between the CAG repeat number and the age of the first symptom onset [9, 10]. In 80% of JHD cases, the mutant *HTT* allele is transmitted paternally [11]. The number of repeats of the family members in both cases was unavailable.

Literature suggests that the most common symptoms of JHD are ataxia, psychiatric disorders, seizures, parkinsonism, dementia, dysarthria, dysphagia, and dystonia [7]. According to the results of our systematic review, depression, suicidal thoughts, dexterity disturbance, and behavior change are also frequent in adolescent-onset patients [11]. In both cases described, the patients developed symptoms from all three main groups: motor, cognitive, and psychiatric. However, the first patient was experiencing more severe psychiatric symptoms; in the second case, motor symptoms were more prominent. The disease affected these patients’ quality of life in different ways.

Researchers have noted that depression, psychosis, attention-deficit/hyperactivity disorder, and anxiety disorders have been identified before the onset of motor symptoms in patients with JHD [12]. Psychiatric disorders in HD are diverse and can occur at any time during the development of the disease, or they can be the first manifestation of it [13, 14]. Studies show that the most common symptom of them all is apathy, which tends to worsen throughout the disease [2, 14, 15]. The occurrence of apathy correlates with the progression of motor and cognitive symptoms in HD and, unlike irritability and depression, indicates disease progression [2, 14]. In both our cases, apathy was one of the first symptoms. Its recognition is essential because apathy affects patients’ motivation to participate in treatment actively. As previously mentioned, bursts of aggression, experienced by the first patient, were especially hard to cope with for the family members. In addition, episodes of psychosis would cause significant distress to the patient himself.

In contrast to the patients with the adult-onset disease, who first experience hyperkinetic symptoms such as chorea and later, after the plateau phase, have hypokinetic symptoms, adolescents with JHD are more prone to hypokinetic motor symptoms, such as bradykinesia, dystonia, rigidity, and balance disturbance, than hyperkinetic ones [2, 3, 12, 13]. Both of our patients were experiencing a gradual decrease in their motor abilities. However, because the expression of motor disorder was different in both patients, the amount and the type of support needed to maximize their independence was also changed.

It has been reported that neuronal degeneration, which is known to be more severe in patients with JHD, results in impaired executive functions, affecting the ability to plan actions and concentrate on an activity [2, 3, 6]. The cognitive symptoms of the disease worsen as the disease progresses, with both declarative and procedural memory deteriorating with time [2]. In the cases presented, cognitive impairments were both early and fast developing. We believe that the loss of cognitive abilities makes it harder for patients and their families to cope with the burden of the disease because it interferes with patients’ ability to receive psychological help and stay motivated for other types of treatment. Inability to acknowledge and appropriately express one’s emotions has a substantial adverse effect on interpersonal communications.

Because the disease inevitably affects the patient’s closest ones, it is crucial to also provide adequate psychological and social support to all the family members. It is essential to mention that, on the basis of studies concerned with finding effective means of help, the best way to support parents, who experience despair and cope with the loss of a healthy child, is through showing the understanding of their current needs and providing consistent support [8, 16]. Last, when consulting family members, special attention should be paid to their well-being. Several studies report that due to permanent physical and emotional fatigue, over time, the caregivers tend to socially isolate themselves and pay less attention to their personal needs, which results in poor treatment results overall [8, 16, 17].

Establishment of multidisciplinary specialist centers for HD, as demonstrated by our experience, not only allows timely diagnosis and treatment plans but also ensures thorough disease management and care for the patients and support for their families, resulting in increased quality of life.

## Data Availability

All data analyzed during this study are included in this published article and were derived from the archived medical records of Vilnius University Hospital Santaros Klinikos.

## Abbreviations

HD: Huntington’s disease
HDCC: Huntington’s Disease Competence Center of Vilnius University Hospital Santaros Klinikos
JHD: Juvenile Huntington’s disease
PCR: Polymerase chain reaction
